# Evaluating the structural reform of outpatient psychotherapy in Germany (ES-RiP trial) - a qualitative study of provider perspectives

**DOI:** 10.1186/s12913-021-07220-7

**Published:** 2021-11-05

**Authors:** Poß-Doering Regina, Hegelow Martin, Borchers Milena, Hartmann Mechthild, Kruse Johannes, Kampling Hanna, Heuft Gereon, Spitzer Carsten, Wild Beate, Szecsenyi Joachim, Friederich Hans-Christoph

**Affiliations:** 1 Department. of General Practice and Health Services Research, University Hospital Heidelberg, Im Neuenheimer Feld 130.3, 69120 Heidelberg, Germany; 2 Department of General Internal Medicine and Psychosomatics, University Hospital Heidelberg, Im Neuenheimer Feld 410, Heidelberg, Germany; 3 Department of Psychosomatic Medicine and Psychotherapy, Medical Center of the Justus-Liebig-University, Friedrichstraße 33, 35392 Giessen, Germany; 4 Department of Psychosomatic Medicine and Psychotherapy, Medical Center of the Philipps University Marburg, Baldingerstraße, 35043 Marburg, Germany; 5 University Hospital Muenster, Section Psychosomatic Medicine and Psychotherapy, Albert-Schweitzer-Campus 1 (Geb. A9), D-48149 Münster, Germany; 6 University Medicine Rostock, Clinic for Psychosomatic Medicine and Psychotherapy, Gehlsheimer Str. 20, 18147 Rostock, Germany

**Keywords:** Evaluation, Qualitative methods, Mental health, Outpatient psychotherapy, Germany

## Abstract

**Background:**

Access to outpatient mental healthcare can be challenging for patients. In Germany, a national structural reform was implemented in 2017 to accelerate and enhance access to outpatient psychotherapy and reduce waiting times. During the first phase of the study ‘Evaluation of a structural reform of the outpatient psychotherapy guideline (ES-RiP)’ and embedded into a process evaluation, the implementation was to be evaluated through assessing general practitioners’ (GPs) and psychotherapists’ (PTs) perspectives regarding utilization of provided new measures, and perceived potential for optimization. Particular focus was on patients with a comorbidity of mental disorders and chronic physical conditions (cMPs).

**Methods:**

This exploratory cross-sectional qualitative study used on-site and online focus group discussions and semi-structured telephone interviews with GPs and outpatient PTs. Generated data were analyzed using thematic framework analysis. Descriptive statistics were used to analyze participant characteristics collected via a socio-demographic questionnaire.

**Results:**

Perspectives on the structural reform were heterogenous. GPs and PTs considered the component of timely initial psychotherapeutic assessment consultations beneficial. GPs disapproved of their deficits in detailed information about the structural reform and exchange with outpatient PTs. Improvement suggestions included structured short information exchange and joint quality circles. The overall number of available outpatient PTs in rural areas was perceived as insufficient. For patients with cMPs, GPs saw patient barriers for therapy access and continuity in low intrinsic motivation, physical impediments and older age. PTs also saw patient challenges regarding low intrinsic motivation and keeping scheduled appointments. They considered post-reform administrative efforts to be high and reported that the regulations (conformity) lead to planning difficulties and financial losses. Reform elements were tailored to fit in with PTs key therapy areas. Stronger networking and joint lectures were suggested as remedy for the currently still limited exchange with GPs. Unlike the GPs, PTs emphasized that accepting patients into psychotherapeutic treatment was independent of a possibly present chronic physical disease.

**Conclusions:**

The findings contribute to understanding the integration of the delivered structural reform into daily care processes and provide an indication about reached targets and potential improvements. Further phases of the ES-RiP study can build on the findings and broaden insights.

**Trial registration:**

Registration-ID DRKS00020344 (DRKS German Register of Clinical Trials.

**Supplementary Information:**

The online version contains supplementary material available at 10.1186/s12913-021-07220-7.

## Background

Around 18 million adults per year in Germany suffer from mental disorders [1, 2]. In addition, about half of them also suffer from a minimum of one chronic physical disease that requires constant monitoring and treatment [3]. Though these patients might have urgent and specific treatment needs, access to outpatient psychotherapeutic healthcare can be challenging for them. However, this group of patients is in particular need of psychotherapeutic care since non-treatment of the mental disorder may lead to a fast deterioration of the physical condition [4]. Long waiting times in outpatient psychotherapeutic care as well as deficiencies regarding interdisciplinary collaboration between therapeutic and somatic care providers affect the access to necessary treatment options additionally.

Subject to the severity of disorder and individual patient preferences and circumstances, mental healthcare is offered in inpatient, day clinic, or outpatient treatment settings where about 30% of patients with mental disorders are treated [5]. Treatment costs are usually covered by the patients’ statutory health insurance plan [6]. The mental healthcare guideline and patient-centered care as regulated by the German statutory health insurers provide the base for all outpatient psychotherapeutic interventions [6, 7].

A structural reform of the psychotherapy guideline (‘Psychotherapie-Richtlinie’) in Germany in 2017 introduced new additional options into the structure of outpatient psychotherapeutic care services. To reduce long waiting times for all patients seeking outpatient psychotherapeutic healthcare and to improve the situation for specific groups of patients, new options for prompt initial diagnostic consultation appointments to assess therapy needs and treatment planning for acute disorders as well as relapse prevention were implemented into the catalogue of the outpatient psychotherapy guideline. In addition, the application processes for both short-term and group therapy were simplified [8]. The reform comprised several key components which were introduced in April 2017: (1) The psychotherapeutic assessment consultation is intended to serve as a timely measure to clarify whether psychotherapy is indicated and which therapy option or further support is appropriate. Assessment consultation uses units of either 25 min or 50 min and is limited to 150 min per adult patient. During the course of assessment consultations, patients receive written information referring to outpatient psychotherapeutic healthcare services and a written feedback form containing the recommendations for further proceedings. Assessment consultations are not part of outpatient psychotherapy consultations as defined in the guideline and thus not part of contingents of allocated psychotherapy units. (2) Psychotherapeutic acute treatment aims to provide a timely intervention after assessment consultations to unburden patients and prevent chronification of mental symptomatology. This option aims for short-term improvement of the symptomatology and states of crisis and emergency. Acute treatment uses either a maximum of 24 consultations (units) of 25 min each or a maximum of 12 consultations (units) of 50 min each which count towards potentially subsequent guideline-oriented outpatient psychotherapy. (3) The option of relapse prevention consultations after intense, longer therapy phases at longer intervals can be applied to stabilize and sustain the impact of the therapy. Depending on the length of the therapy, 8–16 h can be dedicated to relapse prevention. (4) Regional appointment service centers to facilitate timely contact to outpatient psychotherapeutic healthcare services for all patients who seek treatment, regardless of a potential referral. Using the service is optional and patients can still contact PTs directly.

The ES-RiP project aims to evaluate the impact of the implementation of these new care options in general and with a particular focus on patients with cMPs. Prior studies explored single aspects of the structural reform in online surveys with psychotherapists and patients and statutory health insurance billing data. These studies focused on perceptions regarding the use of the new options [9], compared regional as well as urban and rural differences regarding provided outpatient psychotherapeutic healthcare services [3, 10], and assessed waiting times [3, 11, 12]. So far, more differentiated studies have not been conducted to provide insights into potential changes in the care situation of patients with diagnosed comorbid mental and physical disorders after the implementation of the 2017 reform. Currently, there is still a lack of transparency regarding the collaboration between GPs and PTs in outpatient psychotherapeutic healthcare and regarding context-related tailoring of the new care options and their impact on a potential reduction of care access barriers, particularly for the groups of patients with urgent and specific diagnosed treatment needs. Aspects referring to the involvement of GPs are of particular interest since they often are the primary contact for patients and the ones who refer to specialist treatment.

To achieve its goals, the ES-RiP project follows a multi-level approach to investigate and analyze the impact of the new care options from the perspectives of patients, GPs and outpatient PTs. In a sequential explorative approach, a mixed-methods design is used between 2020 and 2022 to evaluate uptake and integration of the new options into daily practice routines, how care providers assess these options, and which potential impact on outpatient care might be attributable. Of particular interest regarding the 2017 reform are whether utilization of outpatient psychotherapeutic healthcare potentially increased in the group of patients with comorbid mental and physical disorders and whether a reduction of waiting times is perceived by care providers in general.

In the first phase of the ES-RiP project in 2020, a qualitative study was conducted on the healthcare provider level using focus groups and semi-structured interviews with GPs and PTs. The aim was to explore their perspectives on the 2017 structural reform and its components and the extent to which the changes brought along by it were realized in general and in particular with regards to the deficits in care for patient groups with chronic somatic co-morbidities.

## Objectives

The objective of this qualitative study was to specifically explore and assess (1) whether GPs and PTs perceived that the structural reform facilitated the inclusion of the targeted individuals, (2) the perceived effects on daily practice of psychotherapeutic healthcare delivery, and (3) the perceived impact of the delivered reform components on access to outpatient psychotherapeutic healthcare for patients with cMPs.

## Methods

### Study design

The ES-RiP study uses a sequential explorative mixed-methods design and a multi-level approach to combine different perspectives, methods and data sources. Thus, the implementation of the reform program will be evaluated on patient, provider and benefactor level. Within a process evaluation, the care provider and patient levels are focused. In complex interventions such as the 2017 reform, a process evaluation aims to understand the functioning of an intervention by investigating the uptake of implemented components, mechanisms of impact and contextual factors [13]. Embedded in the process evaluation in ES-RiP, this present study used a mix of qualitative methods (focus groups and telephone interviews) to explore uptake and perceived impact of the options provided by the reform from the care provider perspectives. Findings of this qualitative study will inform development of a large-scale subsequent paper-based survey targeted at a representative sample of *n* = 1200 GPs and PTs each across several regions in Germany.

### Study population

To be eligible for participation in the qualitative study, GPs and PTs needed to be legally capable, fluent in German, located in the larger area around Heidelberg, Giessen, Munster or Rostock, or work in a practice in or around a major city in Germany, and at best, have practiced in their profession since 2015.

### Recruitment and sampling

The qualitative study followed a structured purposive sampling strategy with regard to region (greater areas around Heidelberg, Giessen, Munster and Rostock, and major cities) to recruit a balanced sample of focus group and telephone interview participants. Recruitment was initiated and carried out by the study team in Heidelberg and supported by the project partners and further cooperating institutions. Written information about the ES-RiP project and the qualitative study, as well as a contact form to be returned to the study team in Heidelberg via fax or e-mail were mailed to potential recruits. Contact details for potential participants were obtained from various sources (publicly available directories listing GPs and PTs in the targeted regions, known contacts in teaching practices, providers referring to in-patient care, professional associations and personal contacts). The contact form provided information on potential dates, times and format of the focus groups. For the telephone interviews, date and time were agreed individually. An informed consent form was sent to all GPs and PTs who stated interest in participation. The study team contacted all interested GPs and PTs by e-mail or phone to provide further detailed information and after receiving the signed consent, date, time and location for the focus group or interview were confirmed. Reimbursement was offered to all participants to compensate for their time with one GP waiving.

### Measures

Tailored questions for the focus groups and the interviews with GPs and PTs covered (a) the uptake and impact of the components of the structural reform as perceived by the participants, (b) consequences of the reform for daily practice in PTs, (c) the perceived impact of diverse context factors on health services for patients in need of outpatient psychotherapeutic care as well as physical chronic care, and additionally, (d) the socio-demographic paper-based questionnaires contained items about participant and practice characteristics.

### Qualitative study

At first, separate on-site focus groups were conducted separately with PTs and GPs by two researchers (experienced Health Services researcher (RPD), Psychologist (MHe)) of the interprofessional study team. On-site focus groups took place in adequate rooms on university campuses and in one case in a hotel conference room. Due to the ongoing Sars-CoV19 pandemic, focus groups were also offered and conducted by the same two researchers using online conference tools. During the on-site focus groups, notes were taken by a member of the respective local study team. In addition to the focus groups, semi-structured guide-based open-ended interviews were conducted with GPs (over telephone) by the Health Services researcher and PTs (via online conference tool) by the researching Psychologist to accommodate participant preferences and to comply with pandemic-related regulations. The interviews were conducted from the researcher’s place of work or home office, and in one case from a university campus.

The focus group and interview guides were developed by the interprofessional team of researchers (Health Services Research, Psychology, Physician) (see Additional file 1 for translated version) and were based on a literature review and pre-defined research questions. All focus group discussions and telephone interviews were audio-recorded, pseudonymized and transcribed verbatim [14]. Focus group discussions also were video-recorded to support transcription. Data was collected until the consistency of findings and deviant observations facilitated the assessment of data sufficiency [15] and code and meaning saturation [16–18].

### Data analysis

All data collected from focus group discussions, interviews, and socio-demographic questionnaires were pseudonymized prior to analysis, electronically saved, and stored on secure servers at the University Hospital, Heidelberg. For the socio-demographic characteristics, descriptive statistics were used to characterize the study sample by tabulating measures of the empirical distribution. According to the level of variables, means, standard deviations (SDs), and absolute or relative frequencies are reported. The statistic software IBM SPSS 25 (IBM, Armonk, NY, USA) was used for tabulation.

**Fig. 1:**
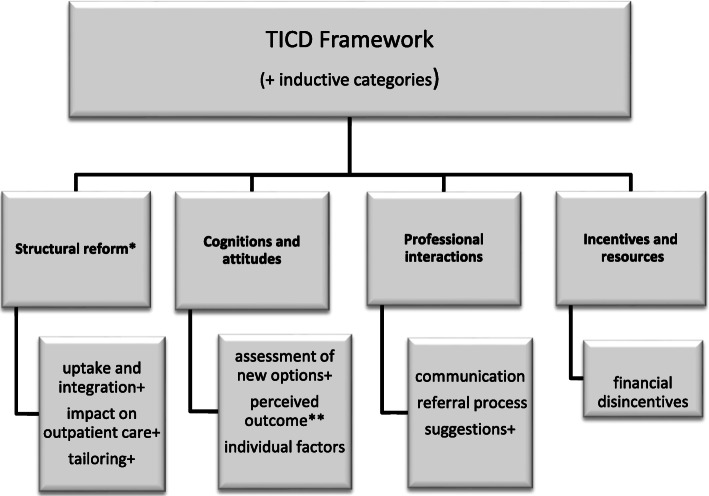
Analytical approach based on the Tailored Implementation for Chronic Disease (TICD) framework. *recoded from TICD domain ‚Recommendation’. **recoded from TICD domain ‘Expected outcome’. +inductive sub-category

Thematic framework analysis [19] was used to classify and organize data deductively according to a priori defined categories derived from domains of the Tailored Implementation for Chronic Disease (TICD) [20] framework to identify determinants of practice which influence current psychotherapeutic healthcare delivery to outpatients. The TICD framework provides seven domains and related sub-domains to support classification of the determinants of an implementation (‘Guideline Factors’, ‘Individual Health Professional Factors’, ‘Patient Factors’, ‘Professional Interactions’, ‘Incentives and Resources’, ‘Capacity for Organizational Change’, and ‘Social, Political and Legal Factors’). Categories were also identified inductively from the generated data. The two researchers who had conducted the focus groups and interviews managed, analyzed, and independently coded the data using qualitative data analysis software (MAXQDA, 2020; Verbi, Berlin). Data coding and analysis were discussed regularly among the researchers to ensure intercoder congruity and to achieve the widest consensus possible. All qualitative data were analyzed until no new themes emerged and the identification of deviant observations and consistency of findings enabled the assessment of data sufficiency and thematic saturation. The two involved researchers had prior experiences with qualitative methods to varying degrees. Pre-defined categories of the TICD were used to identify determinants of practice which influence psychotherapeutic healthcare delivery to outpatients with regards to the components of the structural reform intervention. Reporting of the findings of this qualitative study follows the domains of the TICD and the structure of the analytical framework applied to the data. Quotes extracted from the data are provided. Figure 1 describes the methodical approach and the key categories included in the analysis.

## Results

### Overview

The analysis of the generated data provided in-depth understanding of care provider perceptions referring to the uptake and impact of the structural reform and the perceived significance of the implemented components. Findings are reported in relation to four TICD constructs that emerged as main categories from the analysis: (1) Structural reform (recoded from TICD domain ‘Recommendation’), (2) Cognitions and attitudes, (3) Professional interactions, and (4) Incentives and resources. All other TICD constructs were less prevalent. Included quotes have been translated into English with due diligence and are cited with indication of participant group, mode, number, and transcript position.

In total, seven focus groups with PTs (5 on-site, 2 online), 2 focus groups with GPs (on-site), 1 interview with a PT (online) and 16 semi-structured guide-based telephone interviews with GPs were conducted between July and November 2020. The focus groups had 2–10 participants each, and in total, 21 GPs and 40 PTs participated. Focus groups were carried out within a time frame of 120 min each. Interviews were between 22:35 and 43:59 min with a mean duration of 32 min.

### Participant characteristics

The participants in this qualitative study comprised of GPs (*n* = 21) and PTs (*n* = 40). 67.5% of the PTs were female, their mean age was 54.4 years. 57.5% were psychological PTs, 42.5% were physicians who specialized in PT. Among the GPs, 52.4% were female, with a mean age of 51.9 years (range 36–69). The GPs predominantly had been trained in basic psychosomatic care (90.5%), only 2 GPs were not formally qualified in this subject. 95% of the PTs participated in the focus groups and 76% of the GPS participated in an interview. In both groups, more than half of the participants worked in areas with populations of less than 20,000 people, and only a few in areas with populations of up to 500,000 people.

Table 1 provides details about the socio-demographic and practice characteristics of the qualitative study sample.

**Table 1:** Socio-demographic and practice characteristics of participants in this study (*N* = 61)

Characteristics	GPs (*n* = 21)	PTs (*n* = 40)
Age in years mean (SD)	51.9 (11)	54.4 (9.75)
Sex f (%)	52.4	67.5
Expert years mean (SD)	21 (10.7)	21.4 (10.3)
General Practitioner n(%)	16 (72.7)	
Internal Medicine n (%)	5 (23.8)	
Qualified for basic psychosomatic care n (%)	19 (90.5)	
Psychotherapy (Psychologist)		23 (57.5)
Psychotherapy (Physician)		17 (42.5)
Population at practice location		
< 20,000 n (%)	14 (66.6)	20(50)
< 100,000 n (%)	4 (19)	19 (47.5)
< 500,000 n (%)	3 (14.3)	1 (2.5)
Working full-time n (%)	12 (70.6)	16(64)
Self-employed n (%)	10 (55.6)	37 (92.5)
Single practice n (%)	9 (42.9)	20 (51.3)
Joint practice n (%)	8 (38.1)	19 (48.7)
Professional peer exchange		
once per year n (%)	8 (38.1)	11 (28.2)
once per quarter n (%)	7 (33.3)	9 (23.1)
several times per quarter	2 (9.5)	13 (32.5)
no peer exchange	4(19)	6 (15)
Changes implemented during last 2 years n (%)	18 (85.7)	29 (74.4)

### Structural reform

Uptake of the components and integration into daily practice.

GPs described that they generally provided basic psychotherapeutic care in their practices as needed during routine consultations and offered guidance on how to contact PTs in cases where they deem psychotherapeutic support indicated. During the focus groups and interviews, GPs conveyed that their in-depth knowledge about the structural reform of outpatient psychotherapeutic healthcare was limited to a small number of aspects. They stated they knew about the components introduced in 2017, yet were not familiar with details. The components ‘psychotherapeutic assessment consultations’ and ‘psychotherapeutic acute treatment’ were not clearly separable to them and frequently were considered to be the same component. The option of relapse prevention was largely unknown. While reflecting on the aim and content of the reform during the conversations, the implemented structural components became more transparent to GPs.

‘These were probably all good considerations in this structural reform. But in that respect, maybe that's exactly the issue: The question is, what actually arrives [in daily practice]?’ (FG_GP2_GP1_#55)

PTs were familiar with the details of the 2017 reform, but did not necessarily link them to it. Although there was a high degree of implementation reported, general connotation was critical and associated strongly with growing bureaucracy and less with improving care. One PT considered relapse prevention to be ‘a deceptive package’ (FG5_PT1_#59) as it was part of the approved number of therapy consultations, not an addition. One other PT shared to have used this option ‘quite positively in a number of patients’ (FG_PT5_PT1_#77). When discussing the option of psychotherapeutic acute treatment, PTs stated that it was a part of their daily routines. However, some PTs perceived a gap between necessary and available resources regarding acute treatment, and reported to apply this component not as immediate as intended or not offering it at all. Follow-up therapeutic consultations were not necessarily linked, yet conducted when patient needs were perceived. There were critical and divergent ratings of the assessment consultations, yet positive aspects were acknowledged.

‘From that point of view, assessment consultations are actually something low-threshold and I would see that as quite sensible in terms of care as well.’ (FG_PT2_PT1_#121)

Perceived impact on outpatient care.

Participating GPs did not name any tangible specific effects of the reform on their daily practice routines. They experienced little relief since the reform with regard to patient waiting times and continued to feel obliged to offer bridging work in their own practice when patients faced extended waiting times. However, they perceived that waiting time before the beginning of a therapy was somewhat reduced for some patients, particularly for those with acute psychotherapeutic healthcare needs. It was contemplated that patients with acute symptoms of major depression or anxiety disorders might benefit from the structural reform since they were considered to have been ‘taken care of rather late.’ (GP14_#17).

With regards to patients with comorbid mental and physical disorders, GPs saw prevalent barriers for their access to psychotherapeutic healthcare services that were not reduced by the reform. From their point of view, these barriers ranged from insufficient numbers of available therapists to aspects regarding building and practice structures, as well as scheduling difficulties because of multiple and concurrent necessary treatments. Discussed barriers also comprised patient factors such as missing intrinsic motivation, higher age, and a lower mobility.

‘I have noticed that these initial consultations have been taking place more often since 2017, but the follow-up, the next step is missing. … But that doesn't help the patients much. (GP07_#3)

‘Often it is very difficult to achieve psychotherapeutic insight in a patient. … patients who have a more severe heart disease and other co-morbidities ... they are simply so impaired by the burden of disease that of course they also have psychological co-morbidities. That's absolutely clear. And they're also under-treated in my opinion. But it's also really related to the fact that often the insight is not there.’ (GP08_#30)

PTs did not perceive a general improvement of outpatient care based on the reform and stated that their general recourses, the number of patients, and spectrum of disorders and diseases had not changed. They discussed the potential of reduced waiting times for patients who needed a first telephone contact and an assessment session. However, they also perceived that patients saw several therapists for first assessments, thus limiting access for other patients unintendedly. However, organizational efforts for outpatient assessment and obligatory availability by phone were seen as reducing factors regarding capacities for regular therapy. Fixed time slots for availability by phone were considered to be an obstacle and reducing general capacity, too, since patients and interprofessional partners also called outside the slots. PTs felt irritated by the idea of distinguishing between patients who do have a chronic physical condition and who don’t and did not support this notion. There was acknowledgment of efforts to limit the number of patients with anticipated lower reliability regarding appointments and compensating measures reported such as scheduling these patients for early or late time-slots. In general, anticipated lower reliability was not associated with chronic physical conditions but with patients’ self-regulatory control.

‘I don’t ask for diagnoses, but who calls first...and I think most do so.’ (FG_PT1_PT1_#112)

‘From my point of view, the patients who had to be treated urgently before 2017 already received a therapy place immediately because there is an emergency criterion in place in our practice.’ (FG_PT3_PT6_#45)

### Tailoring

GPs did not describe any tailoring with regards to the elements implemented in the reform since these were not aimed directly at processes in general practice. However, one GP mentioned to occasionally recommend that patients privately paid for therapy consultations outside the statutory health insurance system and apply for refunds with their respective insurer. This was seen as a pragmatic solution when patients in dire need of therapy had to endure waiting times of several months. Another GP mentioned to occasionally write a referral to a psychiatrist ‘in hopes to facilitate the start of urgently necessary therapy’ (GP_10_#35) or a rehabilitation measure.

Elements regulating the processes regarding psychotherapeutic assessment session and PTs’ availability by telephone were generally considered as implemented, but PTs adapted and tailored them to fit to their individual planning and organizational needs. Individual solutions to work around aspects considered as problematic were shared. There was open acknowledgement of efforts regarding ‘how to thread it best’ (FG_PT6_PT4_#69), and of avoiding to offer assessment consultations, but rather schedule appointments with patients who had been on PTs waiting lists for a number of weeks or months already, or only offer assessment consultations when the PT was sure to have free capacities for a therapy as well. PTs who worked in joint practices described that offering assessment consultations and availability by telephone was a lot easier for them since they could split the tasks. Also discussed were aspects related to patient-oriented tailoring by categorizing treatment needs as acute in absence of an actual indication to facilitate a sooner therapy start. PTs described that they felt as if they had to tweak and stretch the existing structure and processes to provide the best possible care to patients, yet they did not feel comfortable with it.

‘I don’t think they [the components] really changed anything significantly for me, I rather adjusted them to fit my previous system as well. ’ (FG_PT3_PT2_#38)

‘I refuse to report availability of assessment consultations or whatever else to the appointment service, because I am quite busy and don’t see why patients should come … for an assessment session to me when I can’t offer a [therapy] appointment. As long as I won’t have to fear massive sanctioning, I will not report availability.’ (FG_PT4_PT5#64)

‘And I always have the feeling I have to keep stretching the system somehow, so to speak, in order to apply for the third therapy, which is actually an ongoing treatment, but I apply for the third therapy. That's stupid.‘ (FG_PT3_PT7_#166)

### Cognitions and attitudes

#### Assessment of new options

Reform components referring to the initial assessment of psychotherapeutic care needs and acute therapy were welcomed by the GPs and considered beneficial for a range of patients. They also saw the simplified option for participation in group therapy as a useful addition. However, no GP reported that one or more of their patients actually was in group therapy, and they did not know of any therapist in or around their catchment area to offer group therapy. After learning about the option for relapse prevention, GPs rated it as very valuable and considered pointing it out to patients to create awareness. The possibility to make an appointment for outpatient psychotherapeutic care via telephonic appointment service centers was discussed controversially. GPs viewed it as problematic with regards to availability and geographic distances between patients domicile and offered place of therapy, or considered it an improvement.

‘I might have two patients who got through and then also got an appointment, but then were not necessarily enthusiastic, because it was further away. And then I've seen at least three who tried to call all the time on several days and really took more than half an hour or three quarters of an hour and didn't get through at all ... . I didn't recommend it to anyone after the negative reports. (GP10_#21)

‘Well, I have the impression that now patients are referred faster. This was more difficult a while ago, I suppose … (GP14_#15)

PTs described that initially they did not welcome the reform but over time implemented components. They acknowledged to be uncomfortable with providing assessment consultations when they were not able to offer a longer term or follow-up perspective and found it difficult to deal with respective patient reactions. The obligatory availability by phone was discussed critically and seen as a limiting factor regarding resources for regular therapy slots. PTs mentioned to frequently neglect this component. The simplified option for group therapy was considered useful.

’ … And all of this unspeakable new stuff with assessment consultations and phone hours and office hours and open hours and whatever, that just gets in the way of care. It just creates difficulties. It would be nice to be able to work in peace.’ (FG_PT5_PT8_#26)

#### Perceived outcome

In general, GPs stated that they did not perceive any essential changes for their patients based on the structural reform of outpatient psychotherapeutic healthcare. They reflected on groups of patients who might benefit more than others and saw patients with less complicated care needs as the ones who could benefit most since they needed shorter consultations and therefore had an improved chance of receiving timely care. At the same time, they stated that exactly these patients could be treated by the GPs themselves to avoid blocking psychotherapy slots for patients with more severe diseases .

‘I think the concept is really good. When I imagine this would work, then my work and the care for my patients could be extremely improved.’ (GP15_#25)

‚ … as long as there are not more psychotherapy slots available, the problem of not receiving therapy in time or on shorter notice is just pushed back, because acute treatment might be fully booked, but outpatient guideline-oriented psychotherapy still has to be waited for. My impression is, if I may critique the restructuring, that this was thought too simple from a political point of view ‘. (GP09_#23)

Congruent with the GPs, PTs also described that they did not ‘… really see such a big difference, honestly‘(FG_PT6_B5_#34) for their patients stemming from the structural reform. The major change they felt was seen as originating in the governance of regulations over their work and organization of it and the unintended result of less availability of therapy hours and an even prolonged waiting time for patients in regular therapy. The structural reform efforts were seen as bureaucracy-orientated re-labeling and not as reformation. However, PTs also reported that patients with a higher functional level most likely might profit from the changes.

‘So basically, at least in our practice, the waiting time for regular therapy slots has definitely gotten longer. That's just the way it is.’ (FG_PT3_B6_#45)

‘Maybe that's a totally distorted impression, but I have the feeling that the functional ones, the work patients, would benefit. So, through the assessment session they can also get to know a practice and then, maybe you are in the right place at the right time and the therapist has an open heart. So that's the way it is, I can't prove it. But I think the patients who have found it difficult to see someone in the past, nothing has changed for them. On the contrary, they might call the appointment service and get an assessment session, but know very well that they will then be forwarded again. When I look at severely depressed patients or social phobics, nothing has changed for them. Except that the therapists have less time because we have to be available by phone and have to offer assessment consultations.’ (FG_PT3_PT5_#58)

#### Individual factors

GPs discussed individual patient factors they considered to influence whether patients actively pursued therapy options. Among those factors were older age, immobility, and deficient German language skills. Also shared were perceptions of patients feeling ashamed when in need of therapy or considering it a weakness. GPs considered themselves to be the first and important point of contact for patients in need of psychotherapeutic healthcare and described providing support within basic psychosomatic care as one of their key tasks in daily practice. They also reflected their own actions critically when contemplating whether psychotherapeutic healthcare options were pointed out strong enough to patients during routine consultations.

’ … people with further chronic diseases tend to be older, more restricted in their potential for self-organization and mobility.’ (GP15_#29)

‘I might have to critically look at myself. Since I am wondering just now, if I might not offer this to certain patients, because I don’t talk about a psychotherapy when I think that it won’t work anyway, because I think, it is not realistic … , the patient can’t get it together or I can’t organize that ...’ (GP15_#33)

PTs stated that chronic physical diseases were no selection criterion when patients inquired about appointment availability by phone. They also described that treating more complicated patients required more time, but was more challenging because of regulations, controlling instances and less compensation. There was concordance in assuming that the proportion of patients who were expected to have difficulties with keeping appointments had to be limited as failures reduced compensation and income.

‘There is a financial incentive to treat in short-term. And there is no incentive for treating difficult patients. You have losses and have to report all the time’. (PT01_#49)

### Professional interaction

#### Communication

GPs and PTs stated that direct communication between them was rare and mainly occurred when they knew each other. Both groups perceived the information flow between them as a one-way-track often containing information regarding different procedures during the care process without a profound professional exchange. They also expressed frustration about certain aspects of existing formal communication which was perceived as insufficient and inadequate. One GP reported that one of his colleagues in the practice was a PT and thus communication and collaboration was effortless. A wide range of aspects referring to communication and exchange of information between the two provider groups were discussed. Among these aspects were content, form and structure of mandatory reporting, and passing on information via the patients.

‘Well I would really like more interaction between GPs and psychotherapists. Because now, it completely runs in a parallel universe.‘ (GP01_#66)

‘ … We might learn where the patient actually was accepted when a consil request arrives and we have to fill it in. Then, we largely don’t hear anything anymore. It is difficult, patients are in therapy over weeks and we basically don’t hear anything about it. (GP12_#3)

‘I know that in the consiliary report, which usually only says “Depression” anyway, I always write “Please with somatic findings”. Would be nice, right? About 10 percent of the consiliary reports are good, they really say something. Mostly only the psychological diagnosis is repeated. Then it says “Depression” in it. So that's total nonsense, it's a waste of paper and time.’ (FG_PT3_PT7#107)

#### Referral process

GPs reported that they encouraged patients to get into contact with PTs when they saw an indication for a specialist diagnosis. They also supported patients with information on therapy options by providing lists of PTs practicing in the area or pointing them directly to PTs they personally knew, but considered it important for the patient to contact PTs themselves. They stated to not distinguish between medical specialists and psychologists when referring patients to therapists, but noted that often they lacked information on the types of therapy methods PTs specialized in. This was considered problematic in acute or complicated case.

‘Sometimes it is difficult to know, also for me as a GP, whom to contact. Then I write an e-mail, then I make a call, then I leave a message.’ (GP06_#35)

PTs acknowledged that direct communication with GPs was a complicated issue for them because of their obligation to confidentiality regarding patient-related information. They reported to frequently use indirect communication via patients. One PT described sending a detailed report once where the GP replied ‘that was the first and only I ever got’ (FG_PT3_PT2_#3). Formal communication via consolatory reports was perceived as insufficient as often required somatic information was not provided by GPs, but ICD-10 coded F-diagnoses were repeated (International Classification of diseases, F00-F99 classification in Chapter V)

‘It is often via patients that I have something delivered or something passed on. And then patients tell me again what the doctor said, … . That is common.’ (FG_PT1_PT4_#4)

### Suggestions for optimization

GPs discussed a variety of suggestions that they perceived could support an optimization of outpatient psychotherapeutic healthcare. Besides the perceived need for more outpatient therapists, GPs phrased they would welcome a more intensive exchange with currently active PTs in their respective region and considered various ways to achieve this. Feedback forms on patients and joint quality circles were frequently mentioned. Also contemplated were options for a more institutionalized professional exchange and types of professional agencies and networks.

‘The institutionalization of a principle regarding patients who are in psychotherapy for more than six months. This would be effective, in my mind, also if it actually was an in-person or video-based consil, because the short reports I got, basically were little meaningful in terms of content.’ (GP01_# 48)

’ … an agency that patients can contact and say ‚I need therapy‘, and the agent is a professional, listens to the patient and says ‚Ok‘, and provides a contact address. Or maybe knows about availabilities so patients don’t have to call 50 therapists each, but a central booking basically.’ (GP04_#60)

PTs appreciated the idea of local practices to support patient placement and emphasized that professional standards could only be granted if these practices would be operated by PTs who also offer therapy. An urgent need was reported for outpatient therapy for patients with posttraumatic stress disorder. Low availability as well as limitations in contingent and regulations were seen as barriers for adequate offers. For patients with physical limitations or during inpatient treatment for physical conditions, PTs saw the need for a permanently installed option of follow-up consultations via phone and video-conferencing as offered during the current pandemic situation.

‘To me it would be a very important consideration whether to demand two or three placement practices in every city and also to support them financially’ (FG_PT7_PT1_#133)

‘And with this clientele in particular, it can be incredibly helpful if you could talk to them over the phone. When someone is in the hospital or when too weak to come.’ (FG_PT5_PT7_#125)

### Incentives and resources

#### Financial disincentives

GPs did not discuss financial aspects in relation to the structural reform since there were no elements included that affected GP practices. General reimbursement of activities within the basic psychosomatic care were contemplated, yet only briefly and as a lesser issue.

PTs acknowledged to be frustrated by this topic ‘therapists do a lot of work for free’ (FG_PT1_PT1_#174). They felt they were not adequately compensated for writing reports, professional interaction, obligatory availability by phone, and even for postage costs incurred with providing obligatory documentation. Offering psychotherapeutic assessment consultations was perceived as a high risk of losing money because ‘people just don’t show up’ (FG_PT3_PT4_# 24). The PTs expressed to be aware of financial incentives associated with guideline-oriented short-term therapy, yet considered this not suitable for patients with long-term needs. They voiced feeling irritated by psychotherapeutic acute treatment for patients in critical situations being compensated less than regular short-term therapy.

‘Yes, but with that you lever out acute therapy’ . (FG_PT1_PT3_#55)

## Discussion

This qualitative study explored the perspectives of both GPs and PTs on a structural reform of German outpatient psychotherapeutic healthcare and provided insights into uptake, reach and fidelity of the implemented restructuring and perceived effects of the intervention components on the daily practice of psychotherapeutic healthcare delivery to outpatients. Of particular interest were aspects regarding the utilization of the implemented measures, the impact on daily psychotherapeutic healthcare for all patients, and in particular, for patients with cMPs. Also focused was the perceived potential for further optimization. Based on selected TICD domains, the systematic analysis of the generated data shows diverse perspectives on the structural reform of outpatient psychotherapeutic healthcare and its impact. The reported findings suggest apparent differences referring to the perceptions of GPs and PTs and the degree of uptake of the intervention components related to the restructuring of outpatient psychotherapeutic care, not to the delivered intervention components itself.

GPs generally knew about the structural reform and the comprised elements but were not familiar with details of all elements. As the first and important point of contact for patients in need of psychotherapeutic healthcare, they provided support, guidance, and also bridged waiting times within the frame of basic psychosomatic care. Putting the patient first in facilitating a prompter and sooner access to outpatient psychotherapeutic healthcare was of utmost importance to them and they strongly supported respective efforts. As they had seen the need for optimization, they effortlessly included pointing out the changes into their daily routines where it seemed appropriate and suited needs and preferences. However, for PTs the adoption of the implemented changes required far more since their work routines had to be reorganized and matched with the new regulation and elements.

Among the most discussed elements of the structural reform were the initial assessment consultations. While GPs largely considered this element to be very beneficial for patients and a first step towards accessing psychotherapeutic healthcare, PTs focused on the organizational and more ethical problems they faced regarding this element. Besides the implied planning and scheduling issues, PTs felt it was inappropriate to hold assessment consultations with patients when they knew they had no capacity available to offer a subsequent therapy. In these cases, the patient had to keep inquiring at PT practices to find an available therapist and re-consult with the GP in the meantime. This troubled the PTs immensely and they found individual ways to avoid offering assessment consultations, thus undermining the structural reform’s intention of providing timely access to psychotherapeutic healthcare. The shift of waiting time being a general issue towards being an issue during the period between initial assessment and actual therapy is an unintended, yet perhaps to be expected outcome of the structural reform. In fragmented, yet complex healthcare settings, organizational change and restructuring efforts are common experiences where leadership, models of care, workforce and governing structures are reshaped in response to legislative and policy changes [21]. Success of such organizational changes often depends on the actors involved, their personal engagement, awareness, and sense of ownership of the change [22]. Efforts of restructuring frequently are based on simplistic notions of organizational change that fail to consider effects of dynamic contexts, individual responses, and agency which makes them unlikely to produce system transformation. Moreover, empirical research on mandated restructuring indicates that beside knowledge and skills, effective organizational change also requires a high level of commitment [23]. However, failing to appropriately implement presumably effective organizational changes such as guidelines or policies might severely limit the potential for patients to benefit from such advances [24]. Changes might be rejected when the primary strategy is to mandate solutions in a top-down approach, the change is not supported and carried by actors such as care providers who can resist or reject, and bureaucracy is encountered [25, 26]. Findings of this qualitative study indicate that the top-down initiated change intended by the structural reform and its components to some extent met resistance of outpatient psychotherapeutic healthcare providers who rejected implementation into their care provision routines and who focused on requirements they perceived as bureaucratic. This indication is substantiated by prior research that supported the same key message: Change is accepted when actors are involved in decisions and activities that affect them, but they resist when it is imposed on them by others and policy mandated change does not get the same weight as clinically driven change [25]. Therefore, change appears to be more likely when there is support and opportunity for interprofessional exchange and collaborative problem-solving, and actors and context are given stronger consideration [27].

One of the key findings of this qualitative study is that PTs perceive no barriers for patients with cMPs and implemented strategies that support them and the patients in pursuing agreed therapy plans even when obstacles are to be faced. However, from the GPs point of view there is still a further need and necessity for improvements, if the target of the structural reform with regards to this group of patients is to be achieved.

To facilitate best care for patients, GPs and PTs as the main providers of outpatient psychotherapeutic healthcare considered it desirable to intensify collaboration and professional exchange between these two groups. Prior research has focused on the perception of collaboration between them and found that PTs considered GPs to be their most important collaboration partners and that collaboration intensity varied from rarely to regularly, with most PTs in favor of more collaboration. A higher intensity of collaboration was linked to small local networks built over time [28]. A recent qualitative study in Norway tested a model for collaborative care. In this model, psychologists and psychiatrists from a community psychotherapeutic health centre were placed in GP practices. When needed, GPs could seek their input or advice and refer patients to them for assessment or treatment. The reported findings document that co-locating GPs and outpatient psychotherapeutic healthcare specialists made them more accessible to each other and enabled detailed, patient-centred case collaboration and mutual learning. The threshold for patients’ access to specialist care was considered to be lowered, treatments could start early, and patient throughput increased. Having the experienced psychotherapeutic health specialists on site facilitated early assessments of symptoms and respective types of treatment required for outpatients, including who could be treated at the GP practice. Thus, care pathways and referral practices could be improved [29]. This is mirrored in the reported experiences of one GP in this present study, even though on a much smaller scale in only one GP practice. A feasibility study conducted in the German primary care setting tested such a collaborative care model under real life daily care conditions. Two large GP practices with a total of 10 GPs provided rooms and workforce capacities to organize the on-site provision of psychotherapeutic assessment consultations by one physician specialist in psychotherapy during regular practice hours once a week over the course of 4 months. GPs reported an unexpectedly high willingness to accept and use the offer and 76% of the approached patients were successfully referred to an initial contact. The specialists also considered the model to be practicable and meaningful, and emphasized that it facilitated to see a broad patient spectrum [30]. A qualitative study conducted with German psychotherapeutic health specialists investigated cross-sectoral collaboration between primary and psychosocial care providers through the implementation of video consultations in primary care. The study reported that participants expected video consultations to improve collaboration with GPs and valued them as a potential means to improve access to outpatient psychotherapeutic health care [31]. In the light of such encouraging findings it appears to be recommendable to test similar models in German outpatient psychotherapeutic healthcare on larger scales.

In this present study, GPs and PTs provided a variety of suggestions on how to achieve intensified collaboration including the use of joint quality circles as platform for informal exchange and joint educational activities. Quality circles are widely used in primary healthcare for quality improvement purposes [32], and are considered to be vehicles for discussions on issues and the reflection of current practice [33]. A strongly advocated suggestion was the institutionalization of collaborative professional networks which are considered to support care coordination and to contribute to improvements in care quality and safety [32]. From the PTs perspective, such coordination would require to be led by psychotherapeutic professionals who are actively involved in therapy processes. Implementation of both suggestions could therefore contribute to improved collaboration between GPs and PTs and optimize the quality of outpatient psychotherapeutic healthcare. A third topic that was repeatedly discussed in the focus groups and the interviews was the number of outpatient psychotherapists and the perception that this number was too small, particularly in more rural areas. In the light of 7.7 million psychotherapeutic assessment consultations in 2019 (up from 4.45 in 2017) and 2.7 million acute therapies (up from 1 million in 2017), efforts to increase the number of available therapists in guideline-oriented psychotherapeutic healthcare could help alleviate the situation [34].

The exploration of perspectives of GPs and PTs in this qualitative study showed that the measures implemented in 2017 were utilized as intended by the structural reform, or tailored to fit the needs of outpatient psychotherapeutic healthcare providers. Yet, the participants did not perceive a strong impact for daily care, and in particular regarding patients with cMPs. However, it is possible that other GPs and PTs do not share the views of the participants in this study or integrated the reform components to a higher degree. Therefore, the findings reported here will serve as basis for a large-scale survey to examine uptake and impact of the structural reform in further detail.

### Strengths and limitations

Analysis in this qualitative study was guided by adequate methodological strategies, and aimed at minimization of bias as well as a reduction of the risk of losing relevant content. Domains derived from the TICD supported the exploration and understanding of facilitators and barriers to the implementation of the components implemented through the structural reform of outpatient psychotherapeutic healthcare in Germany. Reporting of the findings follows the recommendations of the COREQ checklist (COnsolidated criteria for REporting Qualitative research) [35].

Telephone interviews ensured that the additional burden for participants was minimized to the maximum extent. Using a conceptional framework approach ensured considerations of theory driven key themes. This was combined with an inductive approach to identify additional themes. During the process of analysis, a high inter-coder congruence was achieved which reflects a reliable classification and triangulation of the data. The satisfactory number of participants and the density and richness of the generated text data allowed for a thorough analysis of data sufficient to illustrate pre-determined theoretical and inductive categories.

Limitations are apparent in a potential selection bias present in pre-existing participant motivation to disseminate personal attitude and experiences. As inherent in qualitative research, it is also possible that socially desirable answers were given, particularly in the focus group discussions. To limit bias, interviewers established rapport with all participants, asked questions and provided prompts that enabled reflection. Regular debriefing in research team meetings facilitated discussion of perceived tendencies and refinement of adequate approaches throughout data collection and analysis. GPs were not familiar with details of all reform elements and their perception of them might have been not extensive. Participant characteristics might limit the transferability to younger health professionals or other regions.

## Conclusion

This qualitative study contributes to understanding applicability and uptake of the components delivered by the structural reform of the German outpatient psychotherapy guideline. The reported findings indicate different care provider perceptions regarding effects on daily practice and inclusion of targeted groups. They also suggest a potential need for further optimization of outpatient psychotherapeutic healthcare services.

## Supplementary Information

**Additional file 1.** Focus group and interview guide for General Practitioners and Psychotherapists. Evaluation of reorganization of mental healthcare in Germany (ES-RiP).

## Data Availability

All data generated and analyzed during this study are stored on a secure server at the University Hospital Heidelberg, Germany. De-identified sets of the data collected and analyzed during this study can be made available by the corresponding author on reasonable request.

## Abbreviations

cMPs: Comorbidity of mental disorders and chronic physical conditions
ES-RiP: German: Evaluation der Strukturreform der Richtlinien-Psychotherapie (English: Evaluation of the structural reform of outpatient psychotherapy guideline)
GP: General Practitioner
PT: Psychotherapist
SD: Standard Deviation
