# Correction to: Evaluating the structural reform of outpatient psychotherapy in Germany (ES-RiP trial) - a qualitative study of provider perspectives

**DOI:** 10.1186/s12913-021-07290-7

**Published:** 2021-12-02

**Authors:** Regina Poß-Doering, Martin Hegelow, Milena Borchers, Mechthild Hartmann, Johannes Kruse, Hanna Kampling, Gereon Heuft, Carsten Spitzer, Beate Wild, Joachim Szecsenyi, Hans-Christoph Friederich

**Affiliations:** 1 Department. of General Practice and Health Services Research, University Hospital Heidelberg, Im Neuenheimer Feld 130.3, 69120 Heidelberg, Germany; 2 Department of General Internal Medicine and Psychosomatics, University Hospital Heidelberg, Im Neuenheimer Feld 410, Heidelberg, Germany; 3 Department of Psychosomatic Medicine and Psychotherapy, Medical Center of the Justus-Liebig-University, Friedrichstraße 33, 35392 Giessen, Germany; 4 Department of Psychosomatic Medicine and Psychotherapy, Medical Center of the Philipps University Marburg, Baldingerstraße, 35043 Marburg, Germany; 5 University Hospital Muenster, Section Psychosomatic Medicine and Psychotherapy, Albert-Schweitzer-Campus 1 (Geb. A9), 48149 Münster, Germany; 6 University Medicine Rostock, Clinic for Psychosomatic Medicine and Psychotherapy, Gehlsheimer Str. 20, 18147 Rostock, Germany

**Correction to: BMC Health Serv Res 21, 1204 (2021)**

**https://doi.org/10.1186/s12913-021-07220-7**

Following publication of the original article [1], the authors identified an error in the author names of all the authors. The given name and family name were erroneously transposed.

The author group has been updated above and the original article [1] has been corrected.
