# Supplementary material for: Evaluating the structural reform of outpatient psychotherapy in Germany (ES-RiP trial) - a qualitative study of provider perspectives
Source: BMC Health Serv Res. 2021 Nov 5;21:1204. doi: 10.1186/s12913-021-07220-7 (PMC8570230; doi:10.1186/s12913-021-07220-7)
Supplement: Supplementary file 1 — Additional file 1. Focus group and interview guide for General Practitioners and Psychotherapists. Evaluation of reorganization of mental healthcare in Germany (ES-RiP). [file 12913_2021_7220_MOESM1_ESM.docx]

**Additional File 1**

**Focus group and interview guide for General Practitioners and Psychotherapists**

**Evaluation of reorganization of mental healthcare in Germany**  (ES-RiP)

| 1. Collaboration with psychotherapists (daily care routiens)   *Types of collaboration*  *Role of GP*   1. Reorganization of ambulatory mental healthcare   *Associations* |
| --- |
| 1. Knowledge about the reorganization  - *elements* - *dissemination of information* |
| 1. pointing out new options to patients  - *process* - *patient feedback*  1. Impact on daily routines and care  - *challenges* - *perceived changes* - positive changes for patients  1. perspectives on care for patients with both, chronic physical disease and psychotherapeutic care needs  - *challenges* - *impact* |
| 1. context factors  - *structural, organizational, political*  1. added value  - *potential effects* - *unexpected outcomes* - *necessary optimization* |
| - recommendations for future organization of ambulatory mental healthcare   Further ideas   Thank you for participating! |
